# A Family-Wide RT-PCR Assay for Detection of Paramyxoviruses and Application to a Large-Scale Surveillance Study

**DOI:** 10.1371/journal.pone.0034961

**Published:** 2012-04-04

**Authors:** Sander van Boheemen, Theo M. Bestebroer, Josanne H. Verhagen, Albert D. M. E. Osterhaus, Suzan D. Pas, Sander Herfst, Ron A. M. Fouchier

**Affiliations:** Department of Virology, Erasmus Medical Center, Rotterdam, The Netherlands; University of Pretoria, South Africa

## Abstract

Family-wide molecular diagnostic assays are valuable tools for initial identification of viruses during outbreaks and to limit costs of surveillance studies. Recent discoveries of paramyxoviruses have called for such assay that is able to detect all known and unknown paramyxoviruses in one round of PCR amplification. We have developed a RT-PCR assay consisting of a single degenerate primer set, able to detect all members of the *Paramyxoviridae* family including all virus genera within the subfamilies *Paramyxovirinae* and *Pneumovirinae*. Primers anneal to domain III of the polymerase gene, with the 3′ end of the reverse primer annealing to the conserved motif GDNQ, which is proposed to be the active site for nucleotide polymerization. The assay was fully optimized and was shown to indeed detect all available paramyxoviruses tested. Clinical specimens from hospitalized patients that tested positive for known paramyxoviruses in conventional assays were also detected with the novel family-wide test. A high-throughput fluorescence-based RT-PCR version of the assay was developed for screening large numbers of specimens. A large number of samples collected from wild birds was tested, resulting in the detection of avian paramyxoviruses type 1 in both barnacle and white-fronted geese, and type 8 in barnacle geese. Avian metapneumovirus type C was found for the first time in Europe in mallards, greylag geese and common gulls. The single round family-wide RT-PCR assay described here is a useful tool for the detection of known and unknown paramyxoviruses, and screening of large sample collections from humans and animals.

## Introduction

The *Paramyxoviridae* family within the order of *Mononegavirales* includes a large number of human and animal viruses that are responsible for a wide spectrum of diseases [1]. Measles virus (MV) is one of the most infectious human viruses known, and has been targeted by the World Health Organization for eradication through the use of vaccines. The paramyxovirus family includes several other viruses with high prevalence and public health impact in humans, like respiratory syncytial virus (RSV), human metapneumovirus (HMPV), mumps virus (MuV), and the parainfluenza viruses (PIV) [2]. In addition, newly emerging members of the *Paramyxoviridae* family – hendra and nipah virus – have caused fatal infections in humans upon zoonoses from animal reservoirs [3], [4], [5]. In animals, Newcastle disease virus (NDV) is and Rinderpest virus (RPV) was among the viruses with the most devastating impact on animal husbandry. Members of the *Paramyxoviridae* family switch hosts at a higher rate than most other virus families [6] and infect a wide range of host species, including humans, non-human primates, horses, dogs, sheep, pigs, cats, mice, rats, dolphins, porpoises, fish, seals, whales, birds, bats, and cattle [7]. Thus, the impact of paramyxoviruses to general human and animal welfare is immense.

The *Paramyxoviridae* family consists of two subfamilies, the *Paramyxovirinae* and the *Pneumovirinae*. The subfamily *Paramyxovirinae* includes five genera: *Rubulavirus*, *Avulavirus*, *Respirovirus*, *Henipavirus* and *Morbillivirus*. The subfamily *Pneumovirinae* includes two genera: *Pneumovirus* and *Metapneumovirus* [7]. Classification of the *Paramyxoviridae* family is based on differences in the organization of the virus genome, the sequence relationship of the encoded proteins, the biological activity of the proteins, and morphological characteristics [1], [7]. Virions from this family are enveloped, pleomorphic, and have a single-stranded, non-segmented, negative-sense RNA genome. Complete genomic RNA sequences for known members of the family range from 13–19 kilobases in length. The RNA consists of six to ten tandemly linked genes, of which three form the minimal polymerase complex; nucleoprotein (N or NP), phosphoprotein (P) and large polymerase protein (L). Paramyxoviruses further uniformly encode the matrix (M) and fusion (F) proteins, and – depending on virus genus – encode additional surface glycoproteins such as the attachment protein (G), hemagglutinin or hemagglutinin-neuraminidase (H, HN), short-hydrophic protein (SH) and regulatory proteins such as non-structural proteins 1 and 2 (NS1, NS2), matrix protein 2 (M2.1, M2.2), and C and V proteins [1], [7].

Routine diagnosis of paramyxovirus infections in humans and animals is generally performed by virus isolation in cell culture, molecular diagnostic tests such as reverse transcriptase polymerase chain reaction (RT-PCR) assays, and serological tests. Such tests are generally designed to be highly sensitive and specific for particular paramyxovirus species. However, to detect zoonotic, unknown, and newly emerging pathogens within the *Paramyxoviridae* family, these tests may be less suitable. Development of virus family-wide PCR assays has greatly facilitated the detection of previously unknown and emerging viruses. Examples of such PCR assays are available for the flaviviruses [8], coronaviruses [9], [10] and adenoviruses [11]. For the *Paramyxoviridae*, Tong et al. described semi-nested or nested PCR assays to detect members of the *Paramyxovirinae* or *Pneumovirinae* subfamily or groups of genera within the *Paramyxovirinae* subfamily [12]. Although these tests are valuable for specific purposes, nesting of PCR assays and requirement for multiple primer-sets are sub-optimal for high-throughput diagnostic approaches, due to the higher risk of cross-contamination, higher cost, and being more laborious.” Here, a PCR assay is described that detects all genera of the *Paramyxoviridae* with a single set of primers without the requirement of nesting. This assay was shown to detect all known viruses within the *Paramyxoviridae* family tested. As the assay is implemented in a high-throughput format of fragment analysis, the test will be useful for the rapid identification of zoonotic and newly emerging paramyxoviruses.

## Results

### Design of oligonucleotides for PCR detection of paramyxoviruses

**Figure 1:**
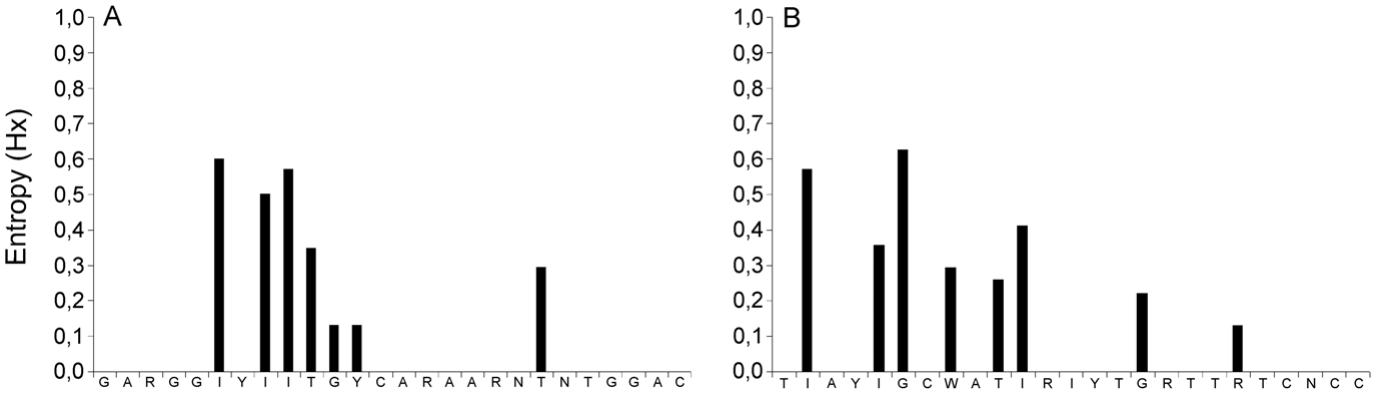
Entropy plots of primer-annealing sites in human and animal paramyxovirus sequences available from Genbank. Primer positions are given in the 5′ to 3′ direction. The sequences recognized by oligonucleotides PMX1 and PMX2 were compared to representative paramyxovirus genera and species sequences (N = 33), and their heterogeneities are displayed in panels A and B, respectively. The degree of heterogeneity was expressed as entropy as defined by Shannon: *H* (1) −∑*f*(*b*, 1) ln [*f*(*b*, 1)], where *H* (1) is the uncertainty at position 1, *b* represents a residue out of the allowed choices for the sequence in question (A, C, G, T, –), and *f*(*b*, 1) is the frequency at which residue *b* is found at position 1 [14], [21].

Thirty-three RNA-dependent RNA polymerase gene nucleotide sequences recorded in Genbank, representative for the species within the *Paramyxoviridae* family, were downloaded. BioEdit Sequence Alignment Editor was used to align the sequences, and to run Clustal W alignments. First, an amino acid sequence alignment was made of all species within the *Paramyxoviridae* family to locate conserved motifs. The most conserved motifs were found in domain III of the polymerase protein. A forced nucleotide alignment was made based on the motifs found in the amino acid alignment. On the basis of this alignment the consensus degenerate and inosine-containing forward and reverse primers, PMX1 and PMX2, respectively, were designed. Because there was ambiguity on several nucleotide positions in the primers, nucleotide degeneracy was applied to both the forward and reverse primer. The primers were designed to amplify a fragment with a total size of 121 base pairs. The variability between the genomic sequences of all paramyxovirus species at each nucleotide position in the PCR primers was calculated using the entropy algorithm available from the Bioedit software package [13], [14]. Entropy values were plotted for each nucleotide position in the primers (Figure 1). Considerable heterogeneity was still present for each of the oligonucleotides, but this was primarily restricted to the 5′ ends of the oligonucleotides. The 3′ ends of oligonucleotides are of greatest importance for the successful amplification by PCR. The 3′ end of the reverse primer PMX2 was designed to anneal to the highly conserved motif GDNQ, which has been proposed to be the active site for nucleotide polymerization [15]. The entropy plots show high conservation of the 14 terminal nucleotides at the 3′ ends of both primers. Forward primer PMX1 contains a thymine at position 20, where hendra virus, nipah virus and PIV-5 contain a cytosine at that position. Reverse primer PMX2 shows 2 mismatches within the 14 nucleotides at the 3′ end of the primer. In this region, APMV-6 and henipaviruses have a single nucleotide mismatch to the primer.

### Optimization of amplification

After primer design, various PCR reaction conditions were optimized, including primer concentrations (range 10 to 50 pmol), magnesium concentrations (range 0.5 to 4 mM), and cycling parameters. For each condition, serial virus dilutions of 10^0^ to 10^7^ were made and conditions were optimized to detect the highest dilution of input virus. Working concentrations of 50 pmol primer yielded the highest sensitivity. The maximum magnesium concentration recommended for AmpliTaq Gold DNA Polymerase of 4.0 mM was found to be optimal in this test. A gradient PCR with annealing temperatures ranging from 38°C to 46°C was performed, with 41°C determined as optimal annealing temperature (data not shown).

### Validation of broad reactivity

**Figure 2:**
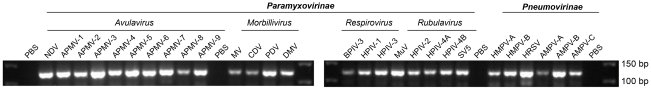
Detection of a wide range of paramyxoviruses by a single RT-PCR reaction. RNA was isolated from the indicated virus stocks and, after cDNA synthesis, used for PCR analysis and subsequent agarose gel electrophoresis. Genomic sequences of twenty-eight paramyxoviruses from six genera were amplified by PCR using the PMX1/PMX2 primer pair. PBS indicates phosphate-buffered saline used as negative control in the entire procedure.

To test the specificity of primers PMX-1 and PMX-2, RNA was isolated from the stocks of 28 paramyxoviruses from 6 genera (Table 1). Viruses from the *Henipavirus* genus were not tested, since these viruses are not available in our laboratory. RNA was isolated from high titer virus stocks in a 50 µL volume, and 11 µL of the RNA was used for cDNA synthesis, PCR amplification and gel electrophoresis to visualize the amplified fragments of 121 base pairs, including the primers (Figure 2).

**Table 1:** Virus stocks tested for evaluation of paramyxovirus detection by RT-PCR.

Virus and classification	Abbrevation	Strain	Passage
*Paramyxovirinae*			
*Avulavirus*			
Avian paramyxovirus type 1	APMV-1	Chicken/N-Ireland/Ulster/2C	Eggs
Avian paramyxovirus type 2	APMV-2	Chicken/California/Yucaipa/56	Eggs
Avian paramyxovirus type 3	APMV-3	Neophema/Holland/449/75	Vero-118
Avian paramyxovirus type 4	APMV-4	Duck/Hongkong/D3/75	Eggs
Avian paramyxovirus type 5	APMV-5	strain Kunitachi	Vero-118
Avian paramyxovirus type 6	APMV-6	Duck/Hongkong/199/77	Eggs
Avian paramyxovirus type 7	APMV-7	Dove/Tenessee/4/75	Eggs
Avian paramyxovirus type 8	APMV-8	Goose/Delaware/1053/76	Eggs
Avian paramyxovirus type 9	APMV-9	Domestic Duck/New York/22/78	Eggs
Newcastle disease virus	NDV	Nobilis ND Clone 30	Eggs
*Morbillivirus*			
Measles virus	MV	Edmonston-Zagreb EZ 19	Vero-118
Mumps virus	MuV	*unknown origin*	*NA*
Canine distemper virus	CDV	Brussels	Vero-118
Phocine distemper virus type 1	PDV	NL/88	Vero-118
Dolphin morbillivirus	DMV	16A	Vero-118
*Respirovirus*			
Bovine parainfluenza virus type 3	BPIV-3	*unknown origin*	tMK
Human parainfluenza virus type 1	HPIV-1	C35	tMK
Human parainfluenza virus type 3	HPIV-3	C243	tMK
*Rubulavirus*			
Human parainfluenza virus type 2	HPIV-2	Greer,VR-92	tMK
Human parainfluenza virus type 4a	HPIV-4A	M-25	tMK
Human parainfluenza virus type 4b	HPIV-4B	CH 19503	tMK
Simian parainfluenza virus 5	SV5	W3	Eggs
*Pneumovirinae*			
*Pneumovirus*			
Human respiratory syncytial virus	HRSV	A2	Hep-2
*Metapneumovirus*			
Avian metapneumovirus type A	AMPV-A	Nobilis TRT	Vero-118
Avian metapneumovirus type B	AMPV-B	Nobilis Rhino CV	Vero-118
Avian metapneumovirus type C	AMPV-C	Colorado	Vero-118
Human metapneumovirus A	HMPV-A	NL/1/00	tMK
Human metapneumovirus B	HMPV-B	NL/1/99	tMK

L-gene fragments of all 28 paramyxovirus strains tested in this experiment were amplified, including members of the *Avula-, Morbilli-, Respiro-, Rubula-, Pneumo- and Metapneumovirus* genera. Additional experiments using RNA isolated from stocks of viruses from different virus families showed no cross reactivity of the assay with influenza A virus, influenza B virus, rhinovirus, human coronaviruses 229E, OC43, and NL63, and adenovirus (data not shown).

### PCR fragment detection using a Genetic Analyzer

**Figure 3:**
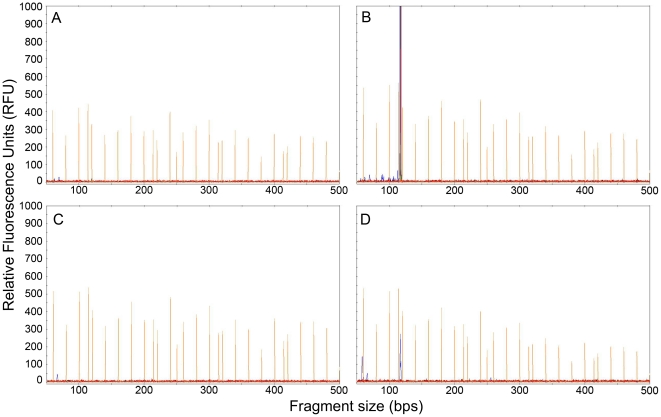
Fragment analysis plots of L gene fragments amplified by RT-PCR. The orange peaks represent LIZ-600 size standards. The size standard peaks are 60, 80, 100, 114, 120, 140, 160, 180, 200, 214, 220, 240, 250, 260, 280, 300, 314, 320, 340, 360, 380, 400, 414, 420, 440, 460, 480, 500, 514, 520, 540, 560, 580 and 600 bps. The blue peaks are PCR products amplified by PMX1 and PMX2, where PMX1 is labelled with the 6-FAM fluorescent dye. A and C represent avian samples in which no paramyxoviruses were found. B and D show peaks around 121 nucleotides. Blue peaks in B and D represent APMV-1 and AMPV-C respectively. The relative fluorescence units are dependent on the amount of amplicon after RT-PCR.

Fragment analysis using a 3130*xl* Genetic Analyzer was used to facilitate screening of larger numbers of samples without the requirement of running agarose gels (Figure 3). To this end, the forward primer PMX1 was labelled with the fluorescent dye 6-FAM. Samples were analyzed in a 96-well format. Seven tenfold serial dilutions of an APMV-3 virus stock were made and amplified using the pan-paramyxovirus RT-PCR assay. Upon agarose gel electrophoresis, 10^−5^ was the last dilution of virus still yielding a visible band. Upon fragment analysis, positive samples could be detected up to a dilution of 10^−7^. When both forward and reverse primers were labelled, no significant increase in detection was observed (data not shown). Therefore, only the forward primer PMX1 was labelled.

### Evaluation of specificity and sensitivity

**Figure 4:**
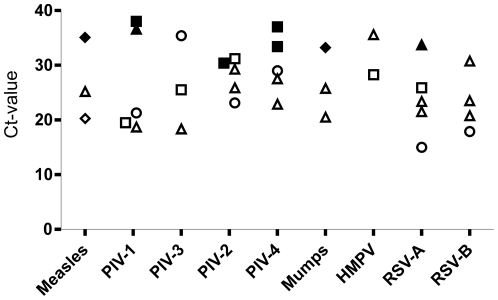
Evaluation of the pan-paramyxovirus RT-PCR assay for virus detection in 35 clinical samples with Taqman-confirmed human paramyxoviruses. Virus loads, as measured by Ct-values in virus specific Taqman assays, are plotted against paramyxovirus species (x-axis). Shapes of symbols indicate the type of clinical specimen (▵; oral samples, □; nose samples, ◊; lung samples, ⋄; other samples). Non-filled shapes represent samples that tested positive in fragment analysis, while filled shapes represent samples that tested negative.

To asses the sensitivity of the PMX-1-PMX-2-based assay as compared to standard diagnostic tests, we obtained anonymized human clinical samples that had tested positive for different paramyxoviruses using specific Taqman assays in the clinical virus diagnostic unit of Erasmus MC. Clinical samples were selected for diversity in virus species, type of clinical specimen, and virus load. The sample collection included clinical specimens positive for MuV, HMPV, MV, PIV-1, PIV-2, PIV-3, PIV-4, RSV-A, and RSV-B. Four different types of clinical specimens were used: oral, (sputum, saliva, throat swab and mouth swab), nose (nose wash and nose swab), lung (broncho-alveolar lavage), and other (plasma and urine). Virus load, as measured by the cycle threshold (Ct) value in real-time Taqman assays, ranged from Ct 15 to Ct 38. Thirty-five samples were selected, RNA was extracted using the MagnaPure LC system, and the RT-PCR assay for detection of paramyxoviruses and fragment analysis was performed (Figure 4). Out of the 35 samples, the pan-paramyxovirus RT-PCR assay detected 27 human paramyxoviruses. The sample with the lowest Ct value (highest concentration target nucleic acid) that remained negative in the pan-paramyxovirus RT-PCR assay was a nose wash containing PIV-2 with a CT value of 30. The virus load in the 8 samples that remained negative in the pan-paramyxovirus RT-PCR assay (mean Ct 34.7, standard deviation 2.5) was lower than the load in the 27 samples yielding a positive reaction (mean Ct 24.5, standard deviation 5.2). Out of the virus specimens tested, 2/3 of the MuV specimens were positive, 4/4 for HMPV, 2/3 for MV, 3/5 for PIV-1, 4/5 for PIV-2, 3/3 for PIV-3, 3/5 for PIV-4, 4/5 for RSV-A, and 4/5 for RSV-B.

Fifty-four additional human throat samples were obtained from the clinical virus diagnostic unit of Erasmus MC. These samples were collected in 2000 and 2001 from patients with respiratory illnesses. The samples tested negative for the presence of RSV, influenza A, B and C, PIV-1 and -4, adenoviruses and rotaviruses by direct immunofluorescence on throat swabs and by immunofluorescence upon tissue culture. Of these 54 samples, one tested positive for PIV-1, two for PIV-4 and three for RSV with the pan-paramyxovirus RT-PCR assay followed by nucleotide sequencing of the PCR fragments (data not shown).

High-titre virus stocks of HMPV, HRSV, HPIV-1, HPIV-2, HPIV-3, HPIV-4, and MV were serially diluted to 10^−10^. Dilutions were tested using agent-specific real-time PCR assays in our diagnostics department. The same RNA was used for the pan-paramyxovirus RT-PCR assay with subsequent fragment analysis. For HMPV, agent-specific real-time PCR assays detected positive samples up to a dilution of 10^−6^, while fragment analysis detected positive samples up to a dilution of 10^−5^. For the other viruses, these comparative dilutions were: RSV 10^−7^ and 10^−5^, PIV-1 10^−7^ and 10^−5^, PIV-2 10^−5^ and 10^−4^, PIV-3 10^−8^ and 10^−5^, PIV-4 10^−6^ and 10^−5^, MV 10^−8^ and 10^−5^. Thus, on average, the pan-paramyxovirus RT-PCR assay was 2-log less sensitive than agent-specific RT-PCR assays.

### Phylogenetic analysis of amplified inserts

**Figure 5:**
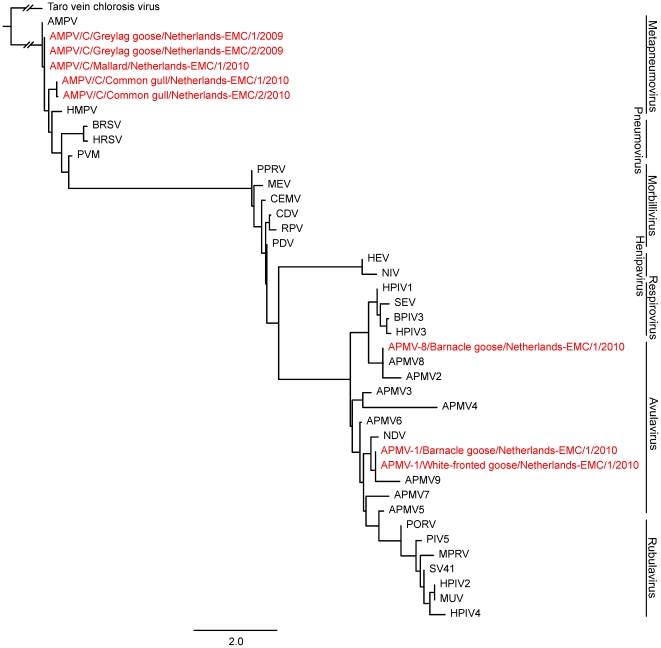
Maximum likelihood (ML) phylogenetic tree based on a 70 nt L-gene fragment for 33 different Paramyxovirus species. An L-gene fragment of the rhabdovirus Taro vein chlorosis virus (AY674964) was used as outgroup. Sequenced in black were obtained from Genbank while sequences in red were obtained from the avian surveillance study reported here. The length of the branch of Taro vein chlorosis virus as an outgroup is broken, and therefore does not represent its genetic distance to the rest of the tree. Accession numbers used for the phylogenetic tree were: NC003443, EU627591, NC009489, NC002200, NC009640, NC006430, NC006428, EU338414, EU403085, FJ177514, GU206351, NC003043, FJ231524, FJ215863, EU910942, NC002617, NC002161, C003461, NC001796, NC001552; NC001906, NC002728, NC001921, NC005283, NC001498, NC006383, Y09630, NC006296, NC001989, NC001781, NC006579, NC007652, NC004148.

The pan-paramyxovirus RT-PCR assay amplifies a highly conserved region in the polymerase gene. To test whether the variability in the amplicon is sufficient to classify virus specimens to a virus subfamily or genus, a phylogenetic analysis of 33 different paramyxovirus L-gene fragments was performed (Figure 5).

As can be seen from the phylogenetic analysis based solely on the 70 base pair nucleotide sequences between primers PMX1 and PMX2 of the paramyxovirus species, the viruses clustered together as genera. These data indicate that the amplicon sequences obtained with PMX1-PMX2 can be used to (at least) roughly classify the viruses detected.

### Application of the pan-paramyxovirus RT-PCR assay to high-throughput detection of paramyxoviruses in avian samples

Samples from migratory birds were collected by expert ornithologists throughout the Netherlands and tested using a real-time RT-PCR assay targeting the influenza A virus matrix gene for an ongoing influenza surveillance program [16]. A selection of influenza virus negative samples was tested for the presence of members of the *Paramyxoviridae* family (Table 2). Samples were selected to span different species of birds, geographical locations, and multiple years. A total of 847 samples were tested for the presence of paramyxoviruses and yielded 8 positives. APMV-1 was found in a barnacle goose (*Branta leucopsis*) and a white-fronted goose (*Anser albifrons*), and APMV-8 in a barnacle goose. Avian metapneumovirus type C (AMPV-C) was found in 5 samples collected from 3 different bird species; mallard (*Anas plantyrhynchos*), greylag goose (*Anser anser*) and common gull (*Larus canus*). To our knowledge, this is the first time that AMPV-C has been found in Europe.

**Table 2:** Avian samples tested for the presence of paramyxoviruses by RT-PCR.

Order	Family	Species	Tested (N)	Positive (N)	N (Percent)	Viruses detected
Anseriforms						
	Ducks					
		Mallard *(Anas platyrhynchos)*	250	1	0,4	AMPV-C
	Geese					
		White-fronted goose *(Anser albifrons)*	224	1	0,5	APMV-1
		Barnacle goose *(Branta leucopsis)*	72	2	2,8	APMV-1, APMV-8
		Greylag goose *(Anser anser)*	30	2	6,7	AMPV-C (2)
		Bean goose *(Anser fabalis)*	15	0	0	
		Canada goose *(Branta canadensis)*	4	0	0	
Charadriiformes						
	Gulls					
		Common gull *(Larus canus)*	84	2	2,4	AMPV-C (2)
	Terns					
		Common tern *(Sterna hirundo)*	2	0	0	
	Waders					
		Dunlin *(Calidris alpina)*	84	0	0	
		Turnstone *(Arenaria interpres)*	30	0	0	
		Oystercatcher *(Haematopus ostralegus)*	18	0	0	
		Common redshank *(Tringa totanus)*	16	0	0	
		Grey plover *(Pluvialis squatarola)*	7	0	0	
		Red knot *(Calidris canutus)*	6	0	0	
		Great ringed plover *(Charadrius hiaticula)*	5	0	0	

Within the avian influenza virus surveillance program, numerous hemagglutinating agents were obtained upon inoculation of 11-day-old embryonated chicken eggs with bird specimens. While the vast majority of these agglutinating agents were identified as influenza A viruses in hemagglutination inhibition assays with antisera against all influenza virus subtypes, seventeen hemagglutinating agents could not be identified as influenza A virus. RNA was extracted from these unknown hemagglutinating agents and the RT-PCR assay for detection of paramyxoviruses was performed. This resulted in the identification of 12 isolates of APMV-1 obtained from mallards, barnacle geese, northern shovelers (*Anas clypeata*), common teals (*Anas crecca*), and white-fronted geese, 1 APMV-4 isolate from a mallard, 2 AMPV-6 isolates from mallards, and 2 APMV-9 isolates from gadwall and mallard.

## Discussion

Here, a high-throughput pan-paramyxovirus RT-PCR assay is described. The polymerase gene is the most conserved gene of the *Paramyxoviridae* family, and was therefore selected as the target for primer design. The primers were designed using an amino acid alignment of the RNA-dependent RNA polymerase protein that contained motifs conserved among all members of the seven genera composing the *Paramyxoviridae* family. The forward and reverse primers anneal at sites that are highly conserved. Sequence comparison of the polymerase gene of paramyxoviruses has revealed six highly conserved domains (I to VI), which are predicted to be essential for the key functions of RNA binding, RNA replication, and protein kinase activity [17], [18]. Domain III was proposed to function as the nucleotide polymerase. The primers used in this assay anneal to domain III. Most interestingly, the 3′ end of the reverse primer PMX2 anneals to the conserved motif GDNQ, which is proposed to be the active site for nucleotide polymerization [15]. Because of this conservation, we can not exclude that this assay may detect other mononegavirales, such as Filo-, Borna-, and Rhabdoviruses. However, Primer PMX1 shows a maximum of 3 mismatches with paramyxoviruses, whilst Filo-, Borna-, and Rhabdoviruses show on average 6 or 7 mismatches with this primer. Likewise, primer PMX2 has a maximum of 4 mismatches with paramyxoviruses, whilst Filo-, Borna-, and Rhabdoviruses show on average 7 to 9 mismatches. It is thus likely that detection of paramyxoviruses is most efficient. In the future, this conserved region may also be used as a target for the development of a pan-rhabdovirus RT-PCR assay or for other members of the order *Mononegavirales* as this region appears to be conserved within several virus families.

Consensus degenerate and inosine-containing primers were used to account for mismatches in strains from different paramyxovirus genera. Using these degenerate primers, all 28 paramyxoviruses that were present in our laboratory, encompassing six out of seven genera, were amplified successfully. Viruses within the *Henipavirus* genus were not tested because these viruses are not available in our facility. The thymine at position 20 in the forward primer does not match the cytosine at the corresponding location in the *Henipavirus* genus L-gene. This same mismatch was also seen in SV5. Since, the primers were able to detect SV5 (Figure 2), detection of henipaviruses should theoretically also be possible.

Application of the pan-paramyxovirus RT-PCR assay to human specimens in which the presence of paramyxoviruses was confirmed using routine species-specific diagnostic tests revealed that the assay detected paramyxoviruses in samples with Ct values up to 35 (Figure 3). Nine different clinical specimen types were tested. Paramyxoviruses were not detected in the two urine samples that were present in the sample set, which was likely due to the low copy numbers present in these samples (Ct 33 and 35). Paramyxoviruses were readily detected in all other clinical specimen types. Overall, the pan-paramyxovirus RT-PCR detected 27/35 paramyxovirus positive specimens from humans and detected paramyxovirus in 6/54 samples that tested negative in routine diagnostic assays. These results appear to indicate satisfactory sensitivity to detect zoonotic, unknown, and newly emerging pathogens within the *Paramyxoviridae* family. It is important to note that the sensitivity of the pan-paramyxovirus RT-PCR assay is less than what can be achieved with more specific RT-PCR based tests.

The application of the pan-paramyxovirus RT-PCR assay was further evaluated using specimens from wild migratory birds. Screening of 847 wild birds in the Netherlands revealed 8 paramyxoviruses, detected using the fragment analysis method. Initially, A SYBR green melting curve-based assay was examined. However, this assay was not sensitive enough for screening of uncultured specimens. Fragment analysis was found to be a robust method for paramyxovirus screening in large sample collections. Three different paramyxovirus species were detected. Among the 8 positive samples, the AMPV-C was detected in three birds. To our knowledge, AMPV-C has not been detected previously in wild birds in Europe. It will be of interest to investigate the prevalence of the European AMPV-C in wild migratory birds further, and to test the genetic relationship with AMPV-C in the USA and HMPV.

Although the primer binding sites for the pan-paramyxovirus RT-PCR assay are highly conserved, the 70 nucleotide amplicon insert displays substantial variation. This variability facilitates the identification of amplified virus sequences to the species or genus level (Figure 4). Thus, the described test not only allows rapid detection of zoonotic, unknown, and newly emerging pathogens, but also gives initial hints to its classification that may aid in the further characterization.

Others have developed primer sets to detect *Paramyxoviridae* viruses previously that have already proven their value [12]. However, one primer set that detects all species of the *Paramyxoviridae* family without the need of nesting PCR reactions has thus far not been described. With the addition of fragment analysis applicable to the RT-PCR assay, paramyxovirus detection has now become possible in a high-throughput manner. The single round pan-paramyxovirus RT-PCR assay described here may thus be a useful tool for the detection of known and unknown paramyxoviruses, and screening of large sample collections from humans and animals.

## Materials and Methods

### Oligonucleotide primer design

Primers were designed to anneal to conserved motifs in the RNA-dependent RNA polymerase gene as described in the results section. Consensus degenerate and inosine-containing primers were designed to account for variability among the different paramyxovirus species. The final optimized forward and reverse oligonucleotide sequences are PMX1 (5′-GAR-GGI-YII-TGY-CAR-AAR-NTN-TGG-AC-3′) and PMX2 (5′-TIA-YIG-CWA-TIR-IYT-GRT-TRT-CNC-C-3′), with G, A, C, T representing normal nucleotides, and I, Y, W, R, N representing Inosine, Pyrimidine (C, T), Weak (A, T), Purine (A, G) and any (A, C, T, G) nucleotide respectively.

### Viral nucleic acid isolation

For initial optimization of virus testing of tissue culture supernatants and allantoic fluids, RNA was isolated manually using the high pure RNA isolation kit (Roche Diagnostics, Almere, The Netherlands) according to instructions from the manufacturer. For high-throughput screening using fragment analysis, RNA was isolated using a MagnaPure LC system with a MagnaPure LC total nucleic acid isolation kit (Roche) according to instructions from the manufacturer.

### Copy DNA (cDNA) synthesis and Polymerase Chain Reaction

A SuperScript III One-Step reverse transcription kit (Invitrogen, Bleiswijk, The Netherlands) was used to synthesize cDNA from extracted RNA. The optimized RT mixture contained 11 µL of RNA extract, 1 µL (500 µg/mL) Random Primers (Promega, Leiden, The Netherlands), 0.5 µL (40 U/µl) Ribinuclease Inhibitor (Promega), and 1 µL (10 mM each) deoxynucleoside triphosphates (Roche) in a 13.5 µL volume. After a 5 min incubation at 65°C for optimal primer hybridisation to template, 4 µL (10×) First-Strand buffer, 1 µL (0.1 M) DTT, 0.5 µL (40 U/µl) Ribinuclease Inhibitor (Promega) and 1 µL (200 U/µL) SuperScript III Reverse Transcriptase was added to the mixture in a 20 µL volume. The RT mixture was sequentially incubated at 25°C for 5 min and 42°C for 1 hour to obtain cDNA.

PCR was optimized with respect to enzymes, primer sets, and concentrations of reagents as well as cycling parameters. The PCR mixture contained 50 pmol of each forward and reverse primer, 4 µL of cDNA, 1 µL (10 mM each) deoxynucleoside triphosphate, 5 µL 10× PCR Gold buffer, 8 µL (25 mM) MgCl_2_, and 0.5 µL (2.5 U/µL) AmpliTaq Gold DNA Polymerase (Applied Biosystems, Bleiswijk, The Netherlands). Water was then added to achieve a final volume of 50 µL. The PCR mixture was incubated at 94°C for 10 min, then 35 cycles at 94°C for 15 s, 41°C for 30 s, 72°C for 30 s, and a final extension at 72°C for 7 min.

### Amplicon detection

PCR products were visualized by blue light after electrophoresis on a 2.5% agarose gel containing 1× GelStar® Nucleic Acid Gel Stain (Lonza, Breda, The Netherlands) in 1× Tris-borate buffer (pH 8.0). A 50 bp DNA Ladder (Invitrogen) was used to estimate amplicon size.

For high-throughput testing, fragment analysis was used. To this end, oligonucleotide PMX1 was labelled with 6-carboxyfluorescein (6-FAM) and PMX2 was used unlabeled. PCR was performed as described above. Subsequently, 0.5 µL LIZ-600 Size Standard (GeneScan, Freiburg, Germany) was mixed with 9 µL formamide and 0.5 µL PCR product. Fragment analysis was performed using the 3130*xl* Genetic Analyzer (Applied Biosystems) and data was analysed using GeneMapper software (Applied Biosystems). Classification of positive human and avian specimens was based on nucleotide sequencing of the PCR fragment and performing a NCBI blastn search on the 70 nt insert.

### Virus stocks and specimens

High titer virus stocks of paramyxoviruses used for validation of broad reactivity of the assay are listed in table 1. Clinical specimens from humans to test for sensitivity of fragment analysis were obtained from the clinical diagnostic unit of the virology department, and were anonymized. Wild birds were trapped by expert ornithologists. Cloacal and/or oropharyngeal swab specimens were collected with sterile cotton swabs. All samples were stored in transport medium consisting of Hanks balanced salt solution containing 0.5% lactalbumin, 10% glycerol, 200 U/mL penicillin, 200 µg/mL streptomycin, 100 U/mL polymyxin B sulfate, 250 µg/mL gentamicin, and 50 U/mL nystatin (ICN, Zoetermeer, The Netherlands). All bird samples were stored at −80°C or at −20°C if rapid transport or storage at −80°C was not possible. Frozen samples were stored at −80°C in the laboratory upon arrival and were thawed no more than two times prior to analysis.

### Phylogenetic tree analysis

Paramyxovirus L-gene fragments spanning the PMX1-PMX2 amplified region were downloaded from Genbank. Nucleotide sequence alignments of paramyxovirus L gene inserts was done using the Clustal W multiple alignment tool in BioEdit Sequence Alignment Editor [13]. Maximum likelihood (ML) trees were inferred using PAUP* (Phylogenetic Analysis Using Parsimony [19], version 4b10), by means of a full heuristic search and the tree bisection-reconnection (TBR) method based on the best-fit models of nucleotide substitution models determined by MODELTEST [20]. The preferred model of nucleotide substitution was GTR+G, based on the Akaike information criterion.

## References

[1] Lamb RA, Parks GD, Fields BN, Knipe DM, Howley PM (2007). Paramyxoviridae.. Fields virology. 5th ed.

[2] Tregoning JS, Schwarze J (2010). Respiratory viral infections in infants: causes, clinical symptoms, virology, and immunology.. Clin Microbiol Rev.

[3] Murray K, Selleck P, Hooper P, Hyatt A, Gould A (1995). A morbillivirus that caused fatal disease in horses and humans.. Science.

[4] Chua KB, Goh KJ, Wong KT, Kamarulzaman A, Tan PS (1999). Fatal encephalitis due to Nipah virus among pig-farmers in Malaysia.. Lancet.

[5] Chua KB, Bellini WJ, Rota PA, Harcourt BH, Tamin A (2000). Nipah virus: a recently emergent deadly paramyxovirus.. Science.

[6] Kitchen A, Shackelton LA, Holmes EC (2011). Family level phylogenies reveal modes of macroevolution in RNA viruses.. Proc Natl Acad Sci U S A.

[7] Fauquet CM, Mayo MA, Maniloff J, Desselberger U, Ball LA (2005).

[8] Scaramozzino N, Crance JM, Jouan A, DeBriel DA, Stoll F (2001). Comparison of flavivirus universal primer pairs and development of a rapid, highly sensitive heminested reverse transcription-PCR assay for detection of flaviviruses targeted to a conserved region of the NS5 gene sequences.. J Clin Microbiol.

[9] Drosten C, Gunther S, Preiser W, van der Werf S, Brodt HR (2003). Identification of a novel coronavirus in patients with severe acute respiratory syndrome.. N Engl J Med.

[10] Ksiazek TG, Erdman D, Goldsmith CS, Zaki SR, Peret T (2003). A novel coronavirus associated with severe acute respiratory syndrome.. N Engl J Med.

[11] Kidd AH, Jonsson M, Garwicz D, Kajon AE, Wermenbol AG (1996). Rapid subgenus identification of human adenovirus isolates by a general PCR.. J Clin Microbiol.

[12] Tong S, Chern SW, Li Y, Pallansch MA, Anderson LJ (2008). Sensitive and broadly reactive reverse transcription-PCR assays to detect novel paramyxoviruses.. J Clin Microbiol.

[13] Hall TA (1999). BioEdit: a user-friendly biological sequence alignment editor and analysis program for Windows 95/98/NT.. Nucleic Acids Symposium Series.

[14] Schneider TD, Stephens RM (1990). Sequence logos: a new way to display consensus sequences.. Nucleic Acids Res.

[15] Malur AG, Gupta NK, De Bishnu P, Banerjee AK (2002). Analysis of the mutations in the active site of the RNA-dependent RNA polymerase of human parainfluenza virus type 3 (HPIV3).. Gene Expr.

[16] Munster VJ, Baas C, Lexmond P, Waldenstrom J, Wallensten A (2007). Spatial, temporal, and species variation in prevalence of influenza A viruses in wild migratory birds.. PLoS Pathog.

[17] Poch O, Blumberg BM, Bougueleret L, Tordo N (1990). Sequence comparison of five polymerases (L proteins) of unsegmented negative-strand RNA viruses: theoretical assignment of functional domains.. J Gen Virol.

[18] Svenda M, Berg M, Moreno-Lopez J, Linne T (1997). Analysis of the large (L) protein gene of the porcine rubulavirus LPMV: identification of possible functional domains.. Virus Res.

[19] Swofford DL (2002).

[20] Posada D, Crandall KA (1998). MODELTEST: testing the model of DNA substitution.. Bioinformatics.

[21] Pierce JR (1980). An introduction to information theory: symbols, signals and noise, 2nd ed.

